# Comparative evaluation of Artec Leo hand-held scanner and iPad Pro for 3D scanning of cervical and craniofacial data: assessing precision, accuracy, and user experience

**DOI:** 10.1186/s41205-024-00245-8

**Published:** 2024-11-25

**Authors:** Samuel D. J. Spears, Thomas Lester, Ryo Torii, Deepak M. Kalaskar

**Affiliations:** 1 Division of Surgery and Interventional Sciences, University College London, London, NW3 2PF United Kingdom; 2 Department of Mechanical Engineering, University College of London, London, United Kingdom

**Keywords:** 3D scanning, Dropped head syndrome, 3D design, MND, Scanning comfort, Scanning accuracy

## Abstract

**Aim:**

This study compares the precision, accuracy, and user experience of 3D body surface scanning of human subjects using the Artec Leo hand-held scanner and the iPad Pro as 3D scanning devices for capturing cervical and craniofacial data. The investigation includes assessing methods for correcting 'dropped head syndrome' during scanning, to demonstrate the ability of the scanner to be used to reconstruct body surface of patients.

**Methods:**

Eighteen volunteers with no prior history of neck weakness were scanned three times in three different positions, using the two different devices. Surface area, scanning time, and participant comfort scores were evaluated for both devices. Precision and accuracy were assessed using Mean Absolute Deviation (MAD), Mean Absolute Percentage Error (MAPE), and Intra-Class Correlation Coefficients (ICC).

**Results:**

Surface area comparisons revealed no significant differences between devices and positions. Scanning times showed no significant difference between devices or positions. Comfort scores varied across positions. MAD analysis identified chin to chest measurements as having the highest variance, especially in scanning position 3. However, no statistical differences were found. MAPE results confirmed accuracy below 5% error for both devices. ICC scores indicated good reliability for both measurement methods, particularly for chin to chest measurements in positions 1 and 3.

**Conclusion:**

The iPad Pro using the Qlone app demonstrates a viable alternative to the Artec Leo, particularly for capturing head and neck surface area within a clinical setting. The scanning resolution, with an error margin within ±5%, is consistent with clinically accepted standards for orthosis design, where padding and final fit adjustments allow for bespoke devices that accommodate patient comfort. This study highlights the comparative performance of the iPad, as well as suggests two methods which can be used within clinics to correct head drop for scanning.

## Background

Head drop is a clinical syndrome otherwise known as dropped head syndrome (DHS) and occurs as a result of the posterior neck muscles weakening. It can be characterised by its “chin to chest” appearance and has several implications both dysphagia, cosmetic and lifestyle. Head drop can be associated with neurodegenerative i.e. motor neurone disease (MND), neuromuscular (i.e. myasthemia gravis), and muscular conditions as well as post-surgical recovery [1]. Treatments for DHS depend on whether it is caused by neuromuscular or non-neuromuscular pathologies, therefore can range from surgical interventions (thymectomy for myasthemia gravis), drug (prednisone for polymyositis), or orthotic (neck collar for MND/ALS). Traditionally people living with MND experiencing head drop are treated using off-the-shelf neck collars, however many do not find them to be suitable [2, 3]. From a survey conducted by Spears et al. (2024) 74% reported they feel the need to wear a collar but only 45% reporting they actually do, and of those 45%, 53% only wear a collar for up to 2h [2, 3]. These neck collars have been developed for use in pre/post-hospitalisation stabilisation of suspected spinal injuries [4, 5]. Whilst current collars do not cause negative clinical implications, they do fall short in meeting the needs of people living with MND with research linking long-term collar use with incidences of pressure ulcers, discomfort and pain for patients [3, 6]. Recent research, including ours, has highlighted the need for a more bespoke solution for patients, one method to achieve this is through the use of 3D scanning and printing [3, 7, 8]. The 3D scanning of the head and neck provides the information on which a bespoke collar can be produced.

3D scanning has been used for body surface scanning in medical research including the following:

1. Photogrammetry: Photos (many) of a subject are captured and used to construct a 3D model.
2. Structured Light 3D scanning: Projection of calibrated light onto the surface of a subject, which then the distorted pattern is captured and used to construct a 3D model.

With a wide variety of options for handheld scanners and techniques, 3D scanning and protocols for use with DHS have not been established prior to this study.

When designing a bespoke neck collar from a 3D scan, the scanning quality has a direct impact on the quality of the collar. Therefore, a suitable 3D scanning technique (device and method) is required that can be used within a clinical setting. Important considerations include price, functionality, accuracy, and usability [9]. Additionally, when clinicians scan a patient with ‘head drop’ to capture their anatomical geometry, several factors must be considered. Firstly, for both patient and user, safety and comfort are important in order to capture a representative model of the patient, especially considering the DHS patient having difficulty in holding their head [9]. Secondly, the ‘head drop’ correction method must offer flexibility in head positioning, as different stages of neck weakness can affect a patient’s achievable range of motion, allowing clinicians to adapt for different patients’ needs [10, 11]. Additionally, duration of scanning and accessibility are important as minimising time spent in clinic not only increases efficiency and resource optimisation, but also reduces waiting times, enhances patient experience [9, 11]. Accessibility is crucial as the method should always be accessible for timely interventions and increased quality of care.

In this current study, there are three aims. Firstly, to compare the precision and accuracy of the iPad Pro versus a more-expensive, specialised scanner (Artec Leo) to capture cervical and craniofacial data for use in the design of a bespoke neck collar. Secondly, to investigate potential techniques which can be used to temporarily correct ‘head drop’ during 3D scanning. Finally, to investigate a simple and reliable scanning protocol that could be used to scan people suffering from dropped head syndrome.

## Methods

### Participants recruitment

18 volunteers (10 females and 8 males) aged 22–57 were recruited for this study. Healthy volunteers were chosen in this initial feasibility investigation to eliminate any clinical complications. The volunteers were postgraduate students and staff members from UCL. Key eligibility criteria were no history of neck weakness and no photosensitive epilepsy. All measurements were taken at the time of scanning. The protocol for this study was approved by UCL Research Ethics Committee (REC) (study reference 20583/001). All methods were performed in accordance with the relevant guidelines and regulations. All participants provided written informed consent.

### Facial dimensions and landmarks

**Fig. 1:**
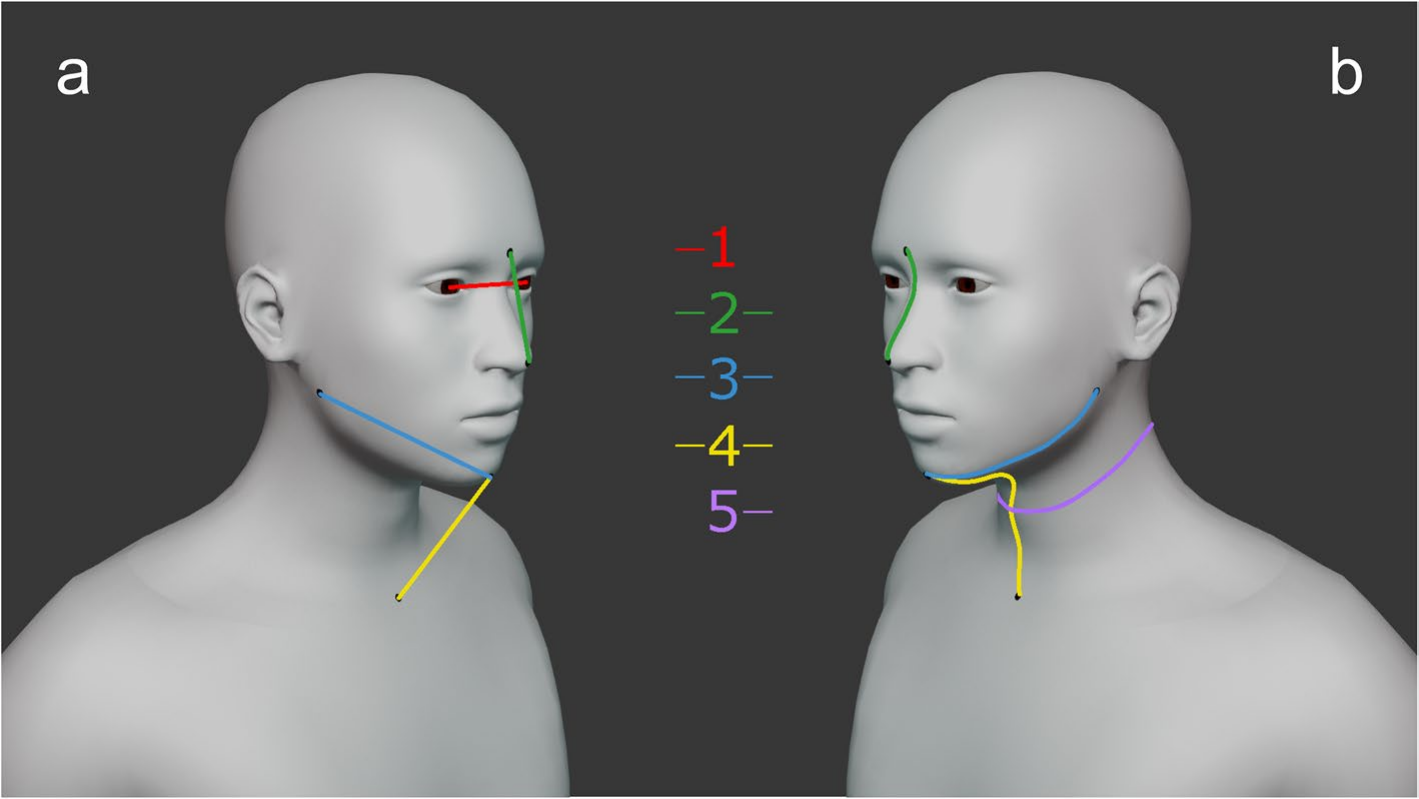
Path of measurement for (**a**) Euclidean measurements and (**b**) Geodesic measurements with landmarks for: 1) interpupillary distance 2) glabella to tip of nose 3) gonion to menton 4) menton to suprasternale notch 5) neck circumference

Five measurements were taken between various facial landmarks including interpupillary distance, nose length, mandible length, chin to chest distance, and neck circumference (Fig. 1). These measurements were taken using two methods: planar (a straight measurement obtained using a 30 cm ruler) and geodesic (a curved measurement following the shape of the feature using a 150 cm tailor’s tape measure). It is important to note that these manual measurements were not directly incorporated into the data analysis but were instead used for secondary comparison. Their purpose was to ensure that the 3D scanning outputs did not deviate significantly from expected anatomical norms. Given this, any potential manual error (± 1 mm) would not impact the overall statistical outcomes of the study, as the primary focus was on comparing the precision and accuracy of the scanning devices themselves. Owing to practical restrictions of the measuring techniques, interpupillary distance was only measured using the planar method and neck circumference was only measured using geodesic.

The definitions of the measurements are as follows:

- Interpupillary distance – distance between two pupils
- Nose length – glabella to tip of nose
- Mandible length – left gonion to menton
- Chin to chest—menton to chest (sagittal plane)
- Neck circumference

Before scanning, black circular self-adhesive dots of 10 mm diameter were placed on the landmarks, which will now be referred to as landmark dots (see also Fig. 1). The glabella was defined as the most anterior point between the bony browridges on the frontal bone. Tip of nose was defined as the most anterior point on the nose. The menton was defined as most anterior point on the edge of the chin. The gonion was defined as the most lateral point on the posterior angle of the jawbone. The suprasternale notch was defined as the inferior point in the notch of the sternum, midway between the clavicles. All definitions were derived from the Anthropometric Survey of U.S. Army Personnel (ANSUR) [12]. The middle of the black dots was used in both manual measurements and those using 3D scanners.

### Head scanning position protocols

**Fig. 2:**
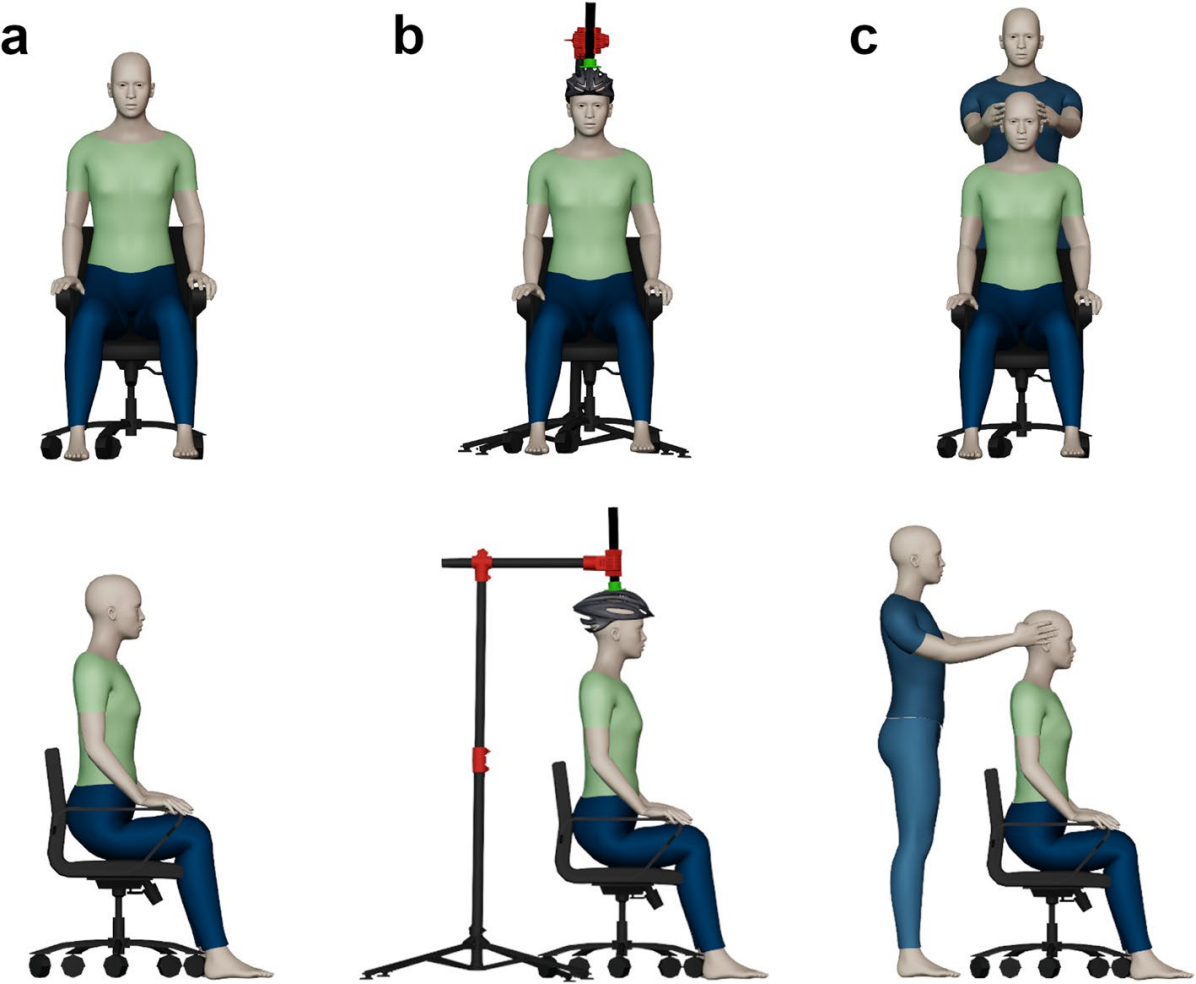
Scanning Positions (**A**) P1 – Natural (**B**) P2 – Assisted with bike helmet & stand (**C**) P3 – Assisted with 2nd experimenter

Three scanning positions were analysed in this study. Position 1 (P1) was used as the baseline position, with participants instructed to sit upright with their head in a natural position with feet planted on the floor and hands resting on their legs (Fig. 2). Natural head position can be defined as a reproducible and standardised position which sees the head in an upright position with eyes fixated on a marked point at a distance at eye level [13]. For position 2 (P2), participants were asked to relax their neck and allow their head to drop forward. A bike helmet with a PVC attachment was placed on their head and a bike stand brought behind the chair they sat on. The participants’ head was then corrected by an assistant to a natural position indicated by the participant, to simulate what would typically be done in clinic. This position was then locked in using the bike stand and attachment. Finally for position 3 (P3), participants were asked to relax their neck and allow their head to drop forward before an assistant corrected the participants head drop manually using their hands.

### 3D Scanners

For this study, two scanning technologies were used. The Artec Leo using a laser-free structured light scanning and the iPad Pro using photogrammetry. The Artec Leo has been used in previous clinical work for 3D imaging of chest walls in patients with anterior chest wall deformities, which aided surgical planning and clinical decision making [14]. The Artec Leo has a 3D resolution up to 0.2 mm, a 3D point accuracy of 0.1 mm, and provides real-time 3D replica images [15]. The point cloud data acquired from Artec Leo is post-processed (triangulation) by Artec Studio software to construct 3D surface models. iPad Pro 12.9 (2021) 5th Gen was selected based on its cost-effectiveness as a viable alternative. Its versatility extends beyond a standalone 3D scanner, making it adaptable within a clinical setting. The iPad has a 12MP wide camera for capturing photos and 4 K video, a 10MP ultra-wide camera for a wider field of view as well as a LiDAR scanning sensor used to capture objects up to 5 m away [16]. The iPad’s large screen allows for clear visualisation whilst scanning and allows for a wide selection of apps and capabilities. Qlone app (version 6.3.5) was chosen for processing captured images for photogrammetry as an affordable alternative with an example application in anatomical study [17]. It utilises both LiDAR scanning and photogrammetry, with LiDAR processing taking place in real time and photogrammetry processing using the online cloud. The Artec Leo scans are imported into Artec Studio to be converted from a point cloud data format to a working 3D object. The Autopilot method was used as this has previously been validated and shown to be more time-efficient method to produce a 3D model than with manual post-processing [18]. The Qlone app eventually exports surface models in standard surface model format, e.g. OBJ, which were imported into Artec Studio as OBJ for consistency when measuring.

### 3D Scanning method and data collection

**Fig. 3:**
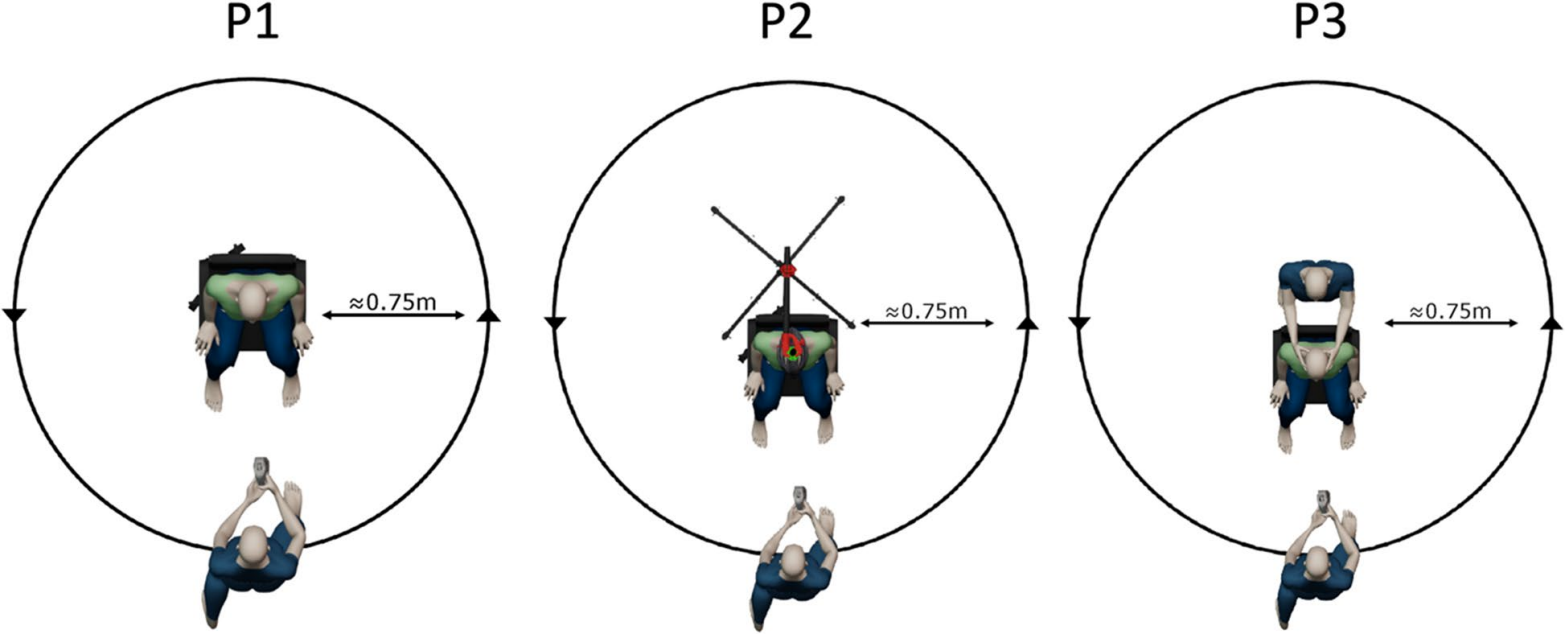
One step scanning procedure

The participants were asked to sit in a chair in a neutral/natural position. The experimenter placed the landmark dots and measured the landmarks using two methods as outlined previously (Fig. 1). The landmark dots were not removed until all the measurements and scans were taken. In case of the landmark dots not adhering due to facial hair, make-up glue was used with participant’s consent. To ensure data collection consistency and accuracy, only one trained experimenter placed the landmarks and performed the measurements. Each participant was then asked to focus on a point at eye level on the wall 2 m away, which was then marked. To ensure consistency, the participant was asked to concentrate on the same point across all scans and positions. Before scanning, each participant was shown both scanners and were informed that the Artec Leo uses a bright white light as part of the scanning process. As part of the eligibility criteria outlined previously, no participant had photosensitive epilepsy. Each participant was asked to confirm whether they were comfortable with keeping their eyes open during the scan and reassured that at no point during the scan would it be pointed directly at their eyes. The scanning procedure began from position 1 through to position 3. For each position, each participant was scanned once with the Artec Leo, allowed a short break, and then scanned with the iPad. This procedure was repeated twice. This routine was used to alleviate any eye strain from the Artec Leo’s light and to reduce operator bias and ensure scan quality. For both scanners, the scan was done in one continuous acquisition for consistency (Fig. 3). For each position, every participant was scanned three times with both scanners, resulting in total 324 scans (three repetitions × two scanners × three positions × 18 participants). Also, the time taken to complete each scan was recorded using a stopwatch by an assistant, which was started from the moment scanning started to when it finished capturing. After scanning, each participant was asked to rate the comfort of each position on a 5-point Likert scale whereby 1 = uncomfortable, 2 = slightly uncomfortable, 3 = neither comfortable nor uncomfortable, 4 = slightly comfortable, 5 = comfortable. There was also an additional comments box where participants could record feedback. Other variables were recorded including file size, number of polygons, surface area, and device specifications. These were noted for transparency and their potential influence on the decision-making process when choosing a 3D scanner.

### Data analysis

**Fig. 4:**
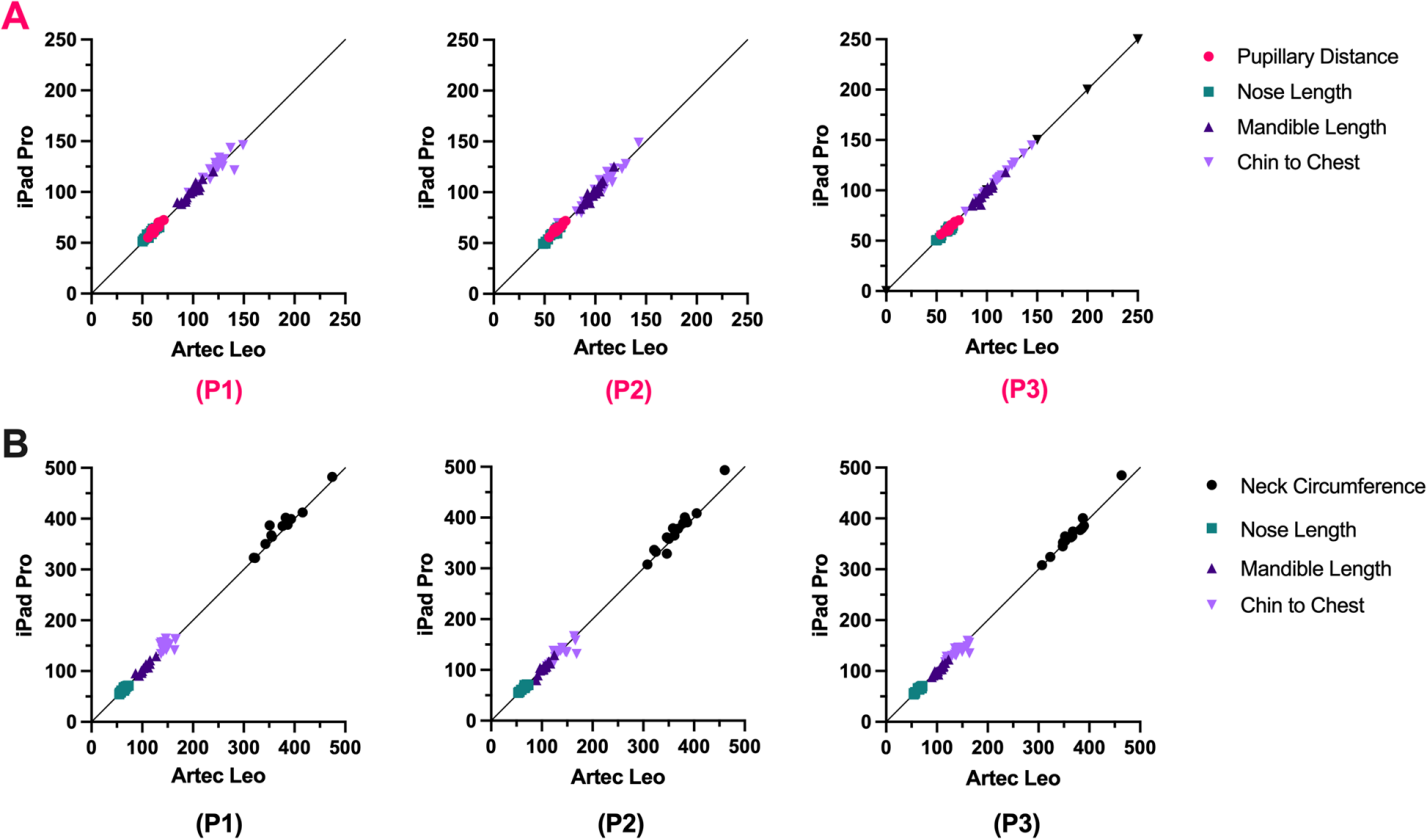
iPad vs Leo QQ plots for (**A**) planar measurements (**B**) geodesic measurements

The independent variables were scanning device and position. Dependent variables were the five measurements, time taken, comfort, and surface area of the head and neck model. A normality test was run on the measurement groups to establish whether the data was parametric or non-parametric (Fig. 4). A paired t-test was performed to evaluate the 3D scanner effect on the surface area captured. A one-way analysis of variance (ANOVA) was conducted to evaluate the effect of position on surface area captured. Time taken was not normally distributed therefore a Wilcoxon signed-rank test was used to evaluate the effect of a device on time-taken for each scan. A Friedman test was used to evaluate the effect of a position on time-taken for each scan. Intra-class correlation coefficients (ICCs) for the five measurements were calculated to understand the agreement between the repeated scans for each scanning technique. ICC’s were calculated using SPSS 29.0 software package. All other statistical analyses were performed using GraphPad Prism 10.1.0 software. The significance level was set a α = 0.05. Additionally, the mean absolute deviation (MAD) between the repeated measurements for the two scanning devices and positions were used to define the repeatability measure for this study (Eq. 1). MAD’s were calculated using measurements calculated in Artec Studio, where both linear and geodesic measurements can be recorded. A larger MAD indicates a lower precision. The mean absolute percentage error (MAPE) was used to calculate the agreement between the two methods of scanning (Eq. 2 & 3). A lower MAPE indicates a higher agreement between the scanners. Artec Leo was considered the ‘gold standard’ with its relatively high instrument resolution (0.1 mm).

1 $$\mathrm{MAD}=\frac{\sum\left|{\mathrm x}_{\mathrm i}-\overline{\mathrm x}\right|}{\mathrm n}$$

2 $${\mathrm{MAPE}}_{\mathrm i}=\frac{{\mathrm{MAD}}_{\mathrm i}}{{\overline{\mathrm x}}_{\mathrm i,\mathrm{Leo}}}\times100$$

3 $${\mathrm{MAPE}}_{\mathrm{average}}=\frac{\sum_{\mathrm i=1}^{18}{\mathrm{MAPE}}_{\mathrm i}}{18}$$

## Results

### Surface area

**Fig. 5:**
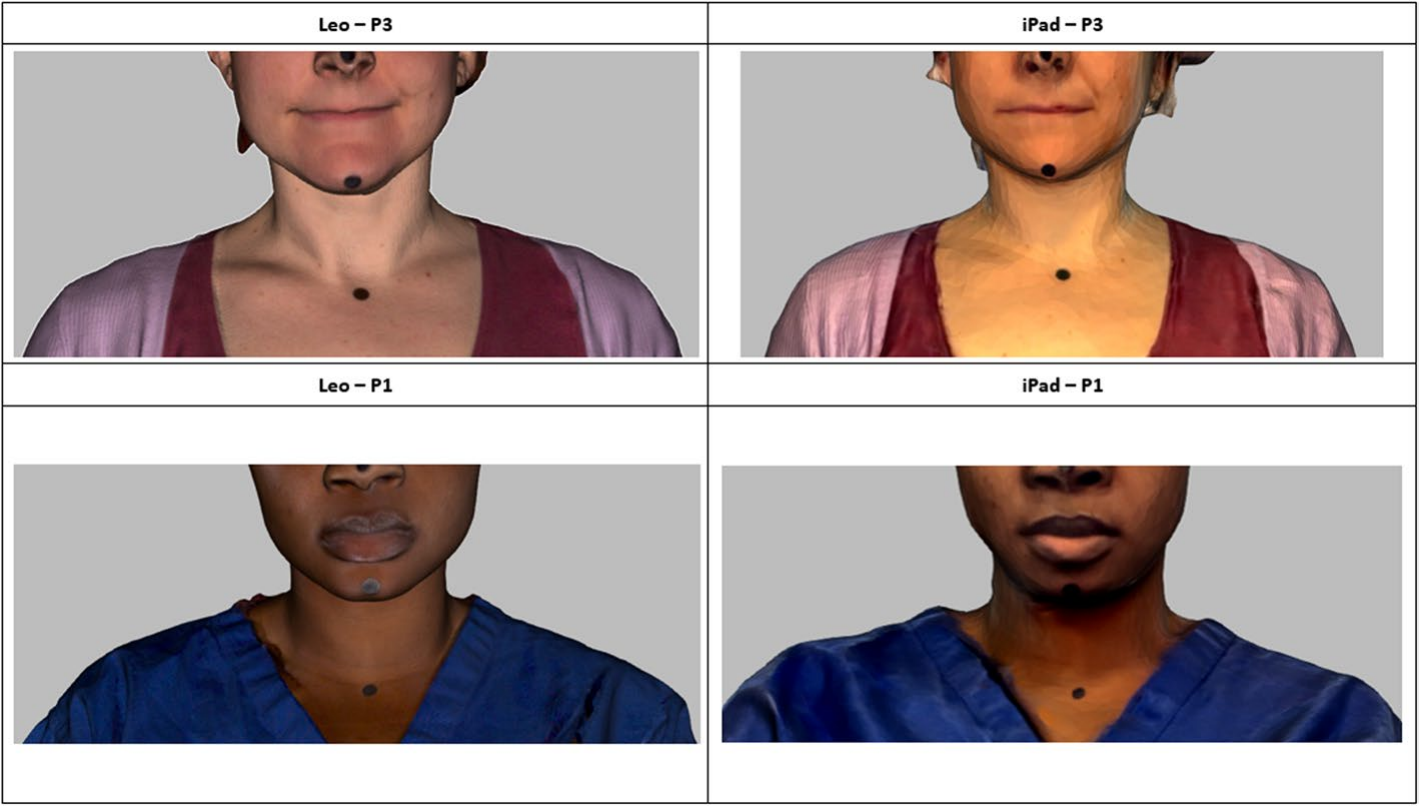
Example of scans captured by each scanning technique

**Fig. 6:**
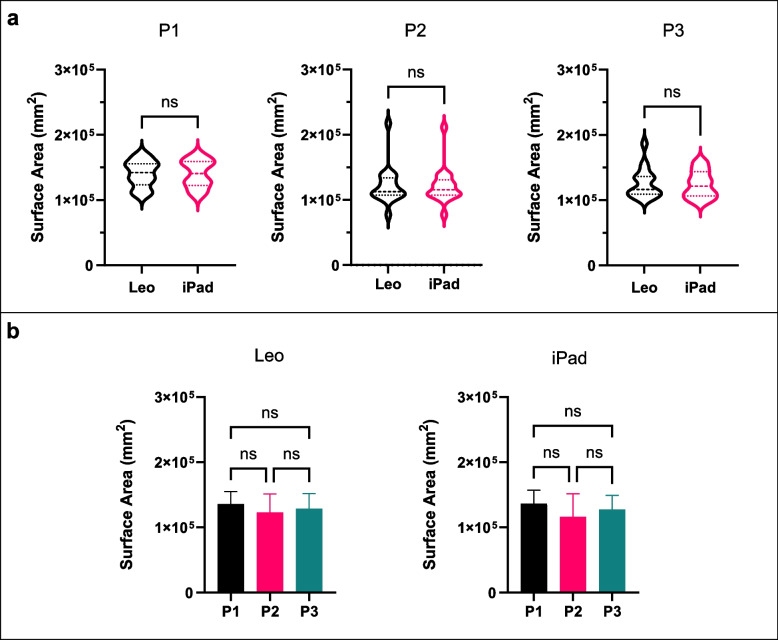
Mean Surface Area (mm2). **A** Comparison between Artec Leo and iPad Pro for P1 (left) P2 (middle), and P3 (right). **B** comparison between positions for both scanners Artec Leo (left) and iPad (right)

Clear, coloured scans were obtained by both scanning techniques (Fig. 5), with all landmark dots clearly visible. The surface area captured by both techniques is presented in Fig. 4. There is no statistically significant difference in the surface area between scanning device or between scanning positions. The paired t-test (Fig. 6– Top) comparing Artec Leo versus iPad is as such; P1 (*p* = 0.798), P2 (*p* = 0.761), and P3 (*p* = 0.645). The ANOVA (Fig. 6 – bottom) results found no significant difference when comparing the effect positions has on surface area captured by each scanning method.

### Time

**Fig. 7:**
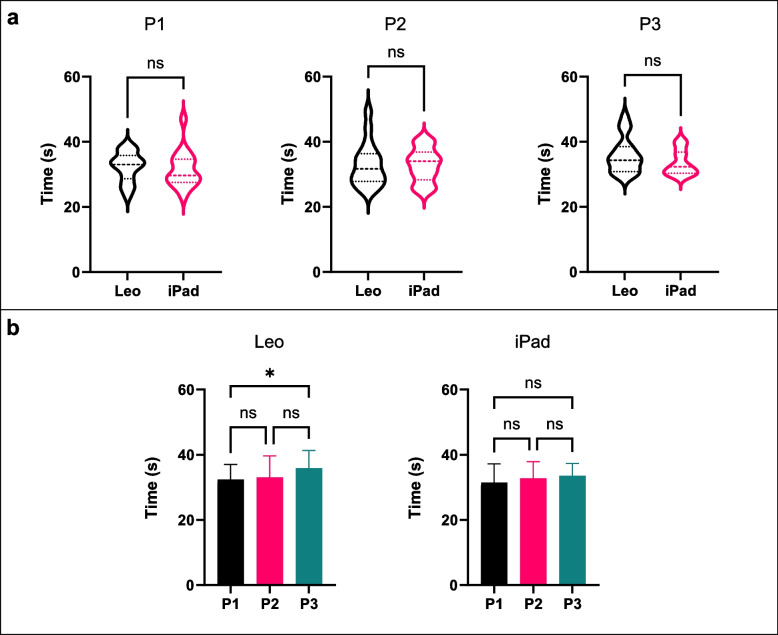
Time taken (s) to perform each scan. **A** Comparison between Artec Leo and iPad Pro for P1 (left) P2 (middle), and P3 (right). **B** comparison between positions for both scanners Artec Leo (left) and iPad (right)

When comparing time taken for each scan against scanning device, there was no statistically significant difference. The Wilcoxon test (Fig. 7 – top) comparing Artec Leo versus iPad was as such; P1 (*p* = 0.323), P2 (*p* = 0.701), and P3 (*p* = 0.178). The Friedman (Fig. 7 – bottom) results found a significant difference for Artec Leo when comparing P1 against P3 (*p* = 0.049). However, comparing all other positions for each scanning device was found not to be statistically significant.

### Comfort

**Fig. 8:**
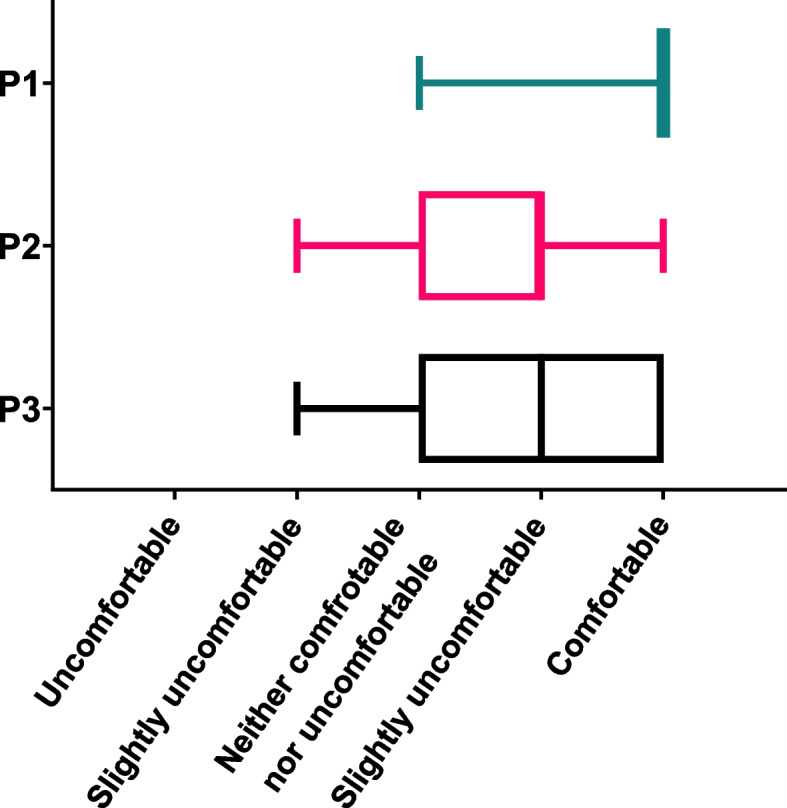
Comfort chart displaying the results of ‘scanning comfort’ for the three positions

Using the feedback forms from each participant, P1 and P3 were rated comfortable, whereas P2 was considered inferior with a rating of slightly comfortable (Fig. 8). P1 was rated the most comfortable with a mean score of 4.8. P2 had the lowest score of 3.6. It was noted in the additional comments section of the comfort assessment that “P3 felt like I had different support at times and it was hard to truly relax.”, “P2 caused slight tension in back but neck position felt comfortable”, “ P3 allowed my head to move about due to sway in assistants arms”, “ P2 helmet felt at times that it may slip off” and, “Leo is slightly uncomfortable due to light”.

### Mean absolute deviation

**Fig. 9:**
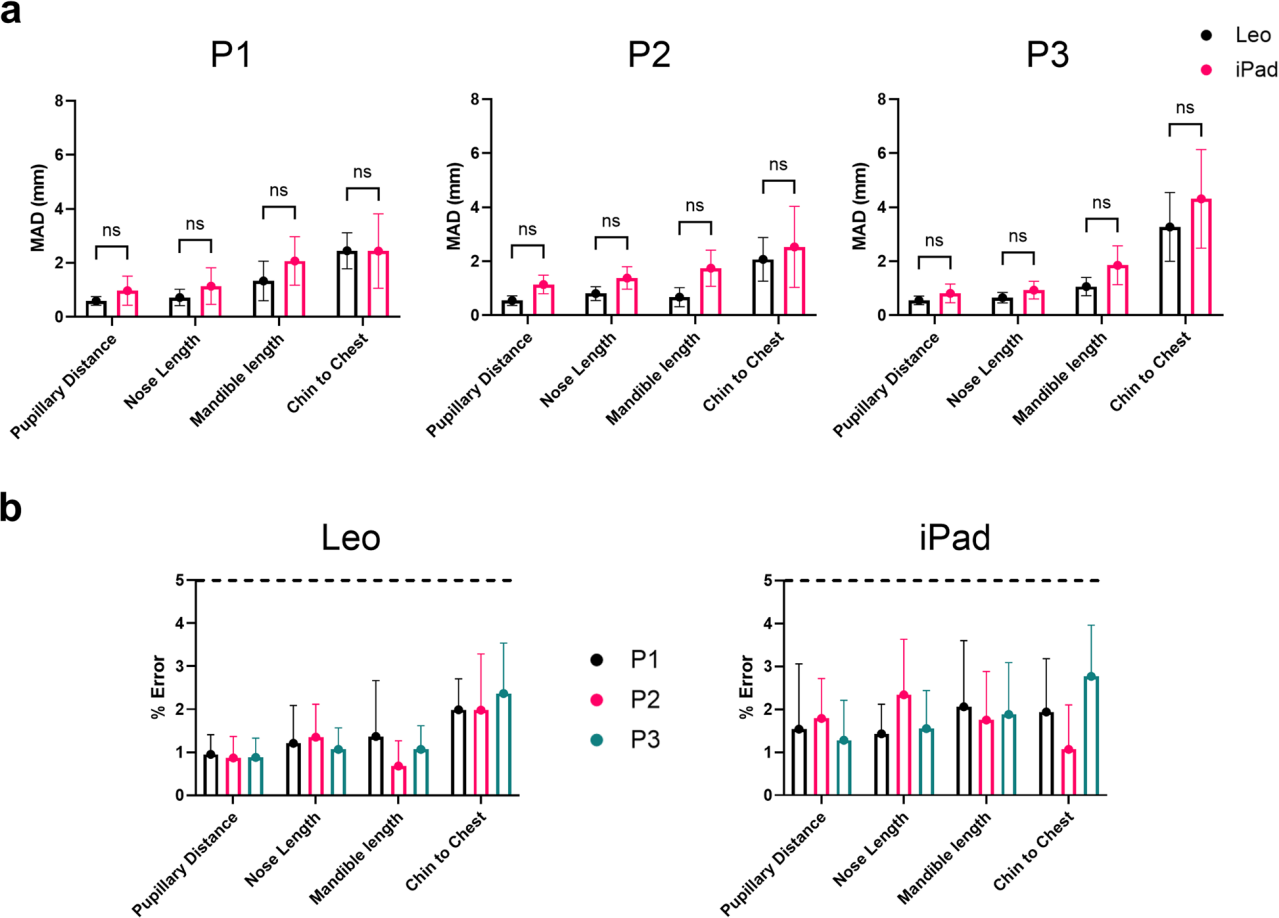
Mean Absolute Deviation (MAD) (mm) for Planar Measurements for both scanners. **A** Comparison between Artec Leo and iPad Pro for P1 (top left), P2 (top middle) and P3 (top right), **B** Comparison for % error between repeat measurements for Artec Leo (bottom left) and iPad (bottom right)

**Fig. 10:**
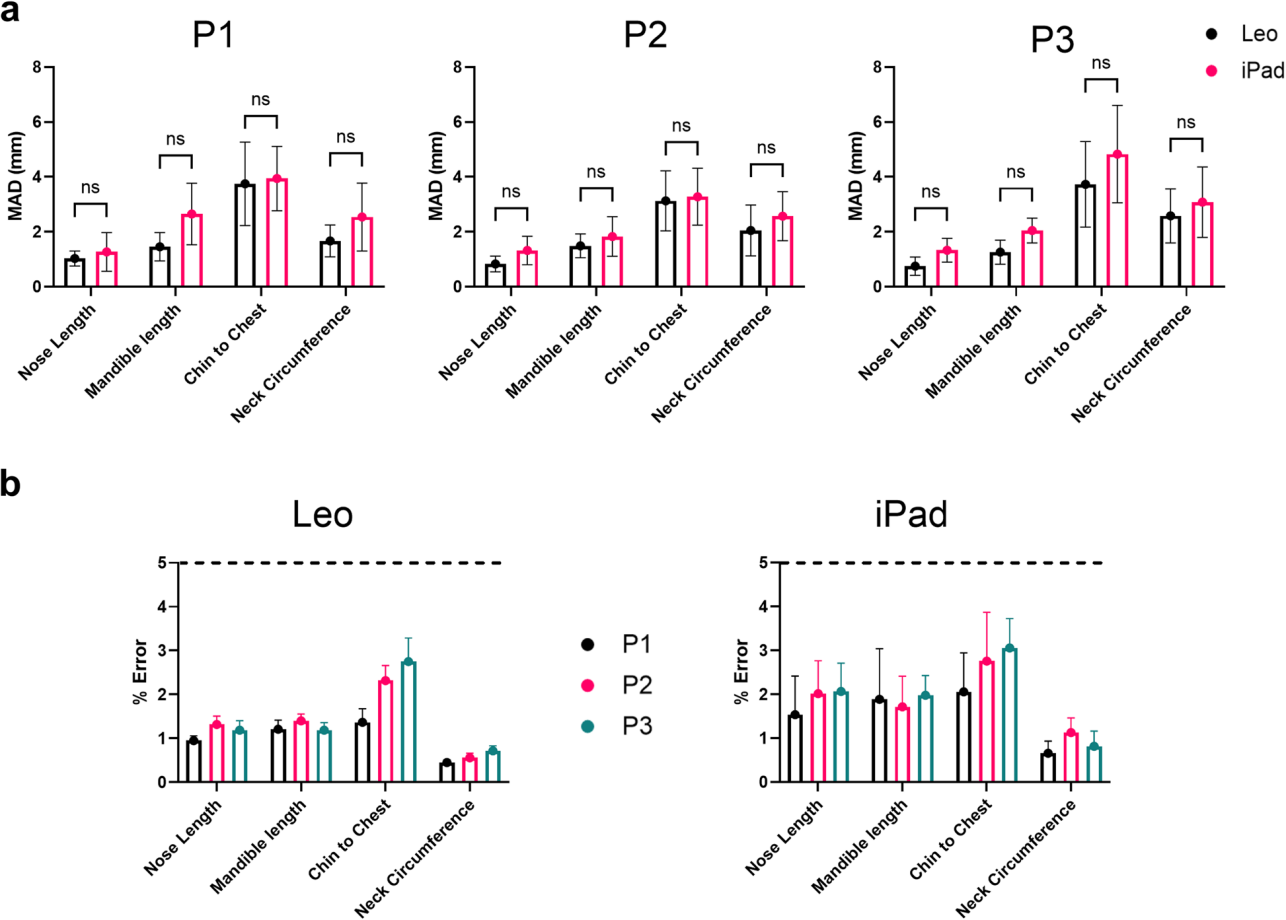
Mean Absolute Deviation (MAD) (mm) for Geodesic Measurements for both scanners. Comparison between Artec Leo and iPad Pro for P1 (top left), P2 (top middle) and P3 (top right) (**B**) Comparison for % error between repeat measurements for Artec Leo (bottom left) and iPad (bottom right)

For evaluation of the scanning precision, the MAD was calculated for the repeat measurements for both scanning devices (Figs. 9 and 10). All MAD values (both planar and geodesic) derived from the models for the 5 measurements where less that 5 mm, which were deemed an acceptable tolerance [7, 19, 20]. Chin to chest had the largest deviation for both scanners for the planar measurements (Fig. 9) across the positions. It also was the largest MAD for the geodesic measurements (Fig. 10) in P3.

### Mean absolute percentage error

**Fig. 11:**
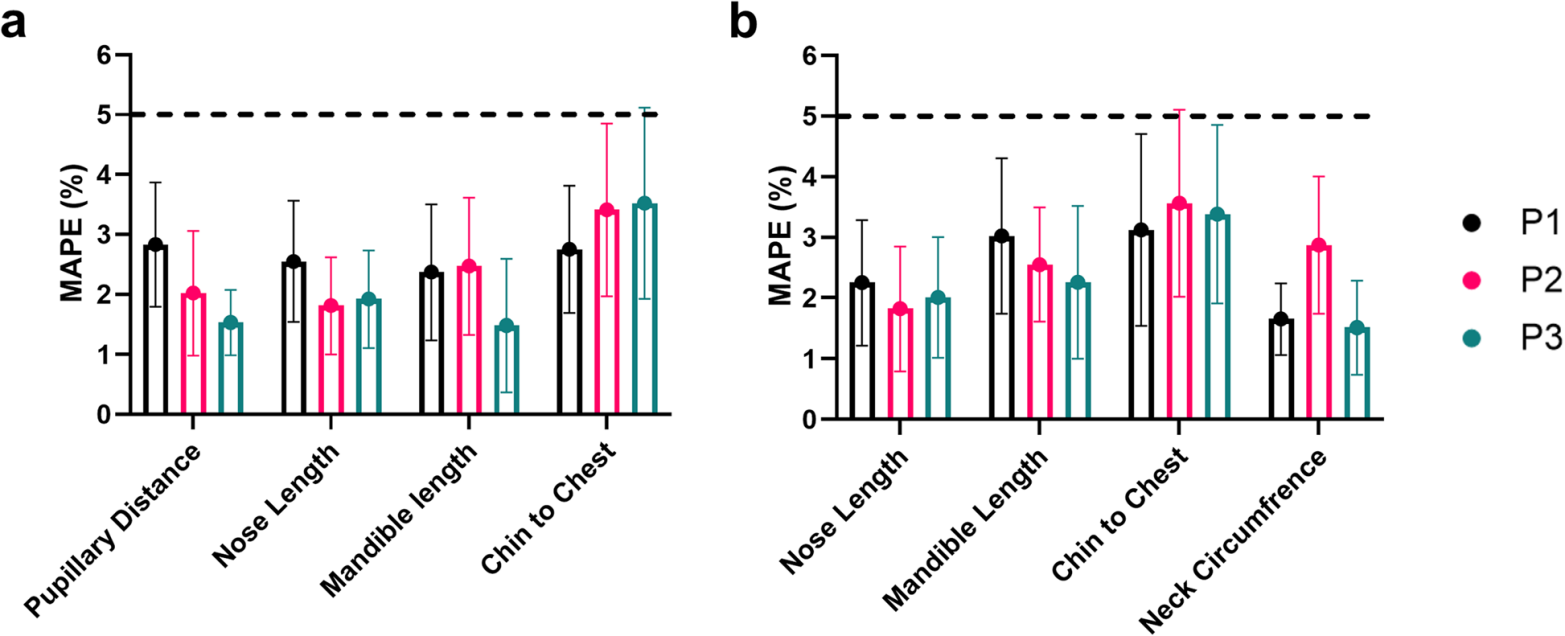
MAPE (%) for iPad against Artec Leo for (**A**) planar measurements (**B**) geodesic measurements

The MAPE was used to calculate the agreement between the iPad and the Artec Leo (Fig. 11). Average differences > 10% were removed as outliers, which resulted from manual measurement error (using mouse to identify measurement points on the software). All MAPEs were below a 5% threshold, which is deemed appropriate for facial measurements [21]. The largest disagreement between the iPad and Leo was the chin to chest geodesic measurements across both measurement techniques. The standard deviation was also largest for chin to chest measurement with planar P3 = 4.76 ± 4.37% and geodesic P3 = 4.85 ± 4.72%.

### Intra class correlation coefficient

The Intra Class correlation coefficient (ICCs) for all five measurements for both planar and geodesic techniques were within a range of 0.81 to 0.99 (as shown in Tables 1 and 2). The worst reliability was found in chin to chest for geodesic.

**Table 1:** Planar ICC's

Dimensions	Artec Leo			iPad		
	P1	P2	P3	P1	P2	P3
Pupillary Distance	0.98	0.99	0.99	0.97	0.91	0.94
Nose Length	0.98	0.98	0.99	0.98	0.95	0.93
Mandible Length	0.97	0.99	0.98	0.98	0.99	0.95
Chin to Chest	0.98	0.99	0.97	0.98	0.99	0.93

**Table 2:** Geodesic ICC's

Dimensions	Artec Leo			iPad		
	P1	P2	P3	P1	P2	P3
Nose Length	0.97	0.98	0.98	0.92	0.96	0.95
Mandible Length	0.98	0.98	0.98	0.93	0.92	0.96
Chin to Chest	0.81	0.97	0.94	0.83	0.96	0.92
Neck Circumference	0.99	0.99	0.99	0.99	0.95	0.99

Finally, additional information which is presented in Table 3. These include the average scan file size (MB), the average polygon count, the percentage of scans containing failed frames, and the number of failed scans. The Artec Leo had the highest average scan size for each position at over 1000 MB for each scan, however, this is reflected in a higher average polygon count at 13,102,106. None of the scans failed with the Leo, whereas 4 of the 162 scans failed to be post-processed into models for the iPad.

**Table 3:** Additional Information

Factors	P1		P2			P3	
	Leo	iPad	Leo	iPad		Leo	iPad
Average Scan File size (MB)	1011.0	4.4	1058.6	4.5		1202.6	4.4
Average Polygon Count	11868148	42162	13437492	45949		14000677	47472
Percentage of Failed Frames (%)	1.9	44	3.7	22		50	37
No. of Failed Scans	0	0	0	1		0	3

## Discussion

The comparison between the Artec Leo and the iPad Pro showed no statistically significant difference in terms of surface area captured between the devices or for positions 1, 2, and 3. This demonstrates that even though different methods were used to scan—structured light for Artec Leo and photogrammetry for iPad Pro using the Qlone app—the use of an iPad with a third-party application is equally effective as a specialised scanner in capturing anatomical features. It should be noted, however, that the Qlone app processed the scans on their secure cloud [22].

Whilst it was not suitable to use the iPad’s LiDAR for this study due to the LiDAR apps being favoured for larger subjects (rooms etc.), the additional use of LiDAR may be beneficial because a study evaluating the iPad Pro’s LiDAR senor for 3D indoor mapping demonstrated that for a surface with a distance less than 1.5 m from the sensor, around 90% of the points are within a distance less than 1 mm from the ground truth [22, 23]. This is superior to our observation, MAD = 5 mm, and well within a clinically acceptable range. A relaxed geometry is often applied when designing bespoke orthoses to allow for a small buffer zone (typically within 5 mm) to ensure comfort and proper fit [7, 18–20]. This buffer helps accommodate padding (typically 5 mm in thickness), which is necessary for patient comfort and does not compromise the functional integrity of the orthosis. Additionally, in clinical settings, final adjustments are made during the fitting process to ensure that the orthosis conforms appropriately to the patient’s anatomy. Several studies [20, 23–26] have investigated the capability of using mobile devices both with LiDAR and photogrammetry to capture facial geometry. These studies found that the use of mobile devices can provide an accessible and affordable way for clinics to introduce best practice methods for scanning and are within a clinically acceptable range of accuracy.

Regarding scanning time, no significant differences were observed between the Artec Leo and iPad Pro for each position. However, there was a statistical difference for the Artec Leo when comparing P1 and 3. This could be attributed to interruptions in the scanning during position 3 caused by navigating around the assistant standing behind the subject and holding the head. It was observed on the Artec Leo’s real-time display that the scan occasionally would fall out of sync at points around the back of the assistant, requiring the scanner to be returned to a previous angle to realign with the previous ‘frame’ before scanning could continue. This may have been caused by the scanner moving too quickly at this point, or possibly due to movement of the assistant. The overall scanning time remained comparable between the two devices across the positions. This highlights the feasibility of both scanning devices and positions in a clinical setting.

The evaluation of the participant comfort scores indicates that of the two corrected positions P3 was more favourable, with P2 scoring the lowest mean comfort score, albeit still within the “comfortable” range. This was likely due to P2 mechanically correcting the “head drop” where it was recorded in feedback that it caused “slight tension in back but neck position felt comfortable” and that at times “felt like the helmet may slip off”. This highlights the impact that the rigidity of a correction method has on participant’s comfort. It is also important to note that this study was completed with healthy volunteers with no history of neck weakness, therefore further research should assess the impact that the type of correction method during scanning has on individuals with head drop. As range of motion may differ from that of healthy participants, care should be taken to avoid potential injury or discomfort.

The MAD for both planar and geodesic measurements showed that the chin to chest measurements exhibited the highest MAD, with the worst being P3. Overall, both devices performed consistently across different positions.

Again, the chin to chest measurement showed the highest variance when analysing the MAPE comparing the iPad Pro against the Artec Leo. This was true for all positions with P3 reporting the worst agreement between the iPad and the Artec Leo. All other errors were below the 5% tolerance threshold, but with the chin to chest measurement for both planar and geodesic, the standard deviation was higher than the 5% threshold.

The ICC showed high agreement above planar measurements for both scanning devices across the three positions (above 0.9). The worst reliability for both the Artec Leo and iPad was the geodesic chin to chest measurement in P1 at 0.81 and 0.83 respectively. A lower ICC was also observed for chin to chest measurement across both measurement techniques for P3 as well, when compared to P2.

This study showed that the measurement with the largest variance across all positions was the chin to chest measurement – apart from P2. At all stages the participant was asked to reset their natural position so; for P1 between each scan the participant was allowed to relax and then reset to an NHP for the next scan, this was also true for P3. This shows that there is a natural range of motion for head position. Therefore, when correcting head drop for people living with MND great care should be taken when trying to find the “natural” head position (before DHS) and should be one that is comfortable to the patient and possible taken several times to find an averaged comfortable position. This is important as scans are used in the design process for bespoke orthosis, therefore surface area captured, head to chin distance and accuracy of neck circumference could impact whether the bespoke orthosis will be accepted or rejected due to fitting/comfort.

There was a significant difference between the file size for each scan captured by the Artec Leo and iPad Pro. The average file size for the iPad was 4.4 MB whilst the Artec Leo was over 1 GB. This may be of consideration to clinicians and rehabilitation engineers in storing patient data. Also noted in this study that the iPad did have a number of failed scans (3/162) where the Artec Leo did not. This is a consideration for potential users in the possibility that scans may fail and thus are required to be redone. Training for both scanning devices would be required and recommended by the experimenter as each position required slight adjustments for both scanners. Another worthy note is that across the scans the Artec Leo and iPad Pro contained a number of scans with failed frames. For the Artec, those frames were removed in postprocessing which ensured a smooth final model, whereas for the iPad, using Qlone’s app, the postprocessing is done on their servers whereby there is no control to remove these ‘frames’, so are reflected directly in the final model as missing elements. This should be taken into consideration when choosing a scanning device.

The results from this study indicated that the iPad Pro is a viable alternative to the Artec Leo for scanning the craniofacial/cervical region. Our study also showed that both scanners consistently achieved errors well below 5%, which aligns with these accepted standards in orthotic design. Therefore, the resolution provided by these scanning tools is indeed sufficient for producing bespoke neck orthoses that may require minor adjustments during the fitting process to achieve optimal comfort and functionality.

### Limitations

The use of healthy volunteers in this study does not represent the anatomical variations in individuals suffering from pathologies affected by head drop. The method of 3D scanning used by the iPad is dependent on the app chosen. There are third-party applications out there that utilise the LiDAR sensor inbuilt in the iPad Pro, however at time of this study, these apps mainly focus on larger captures i.e. rooms, and weren’t found to be optimised for anatomical capture.

## Conclusion

In conclusion, this study suggests that the iPad Pro is a viable alternative in capturing head and neck data across various positions. This study highlights the comparative performance of the iPad, as well as suggests two methods which can be used within clinics to correct head drop to be able to scan. This contributes valuable information for clinicians and researchers in choosing appropriate scanning tools for specific applications.

## Data Availability

Data is provided within the manuscript or supplementary information files.

## Abbreviations

ALS: Amyotrophic Lateral Sclerosis
ANOVA: Analysis of variance
ANSUR: Anthropometric Survey of U.S Army Personnel
DHS: Dropped Head Syndrome
ICC: Intra Class Correlation Coefficient
MAD: Mean Absolute Deviation
MAPE: Mean Absolute Percentage Error
MND: Motor Neurone Disease
NHP: Natural Head Position
REC: Research Ethics Committee
UCL: University College London
